# The fight to keep resistance at bay, epidemiology of carbapenemase producing organisms (CPOs), vancomycin resistant enterococci (VRE) and methicillin resistant *Staphylococcus aureus *(MRSA) in Norway, 2006 - 2017

**DOI:** 10.1371/journal.pone.0211741

**Published:** 2019-02-04

**Authors:** Petter Elstrøm, Elisabeth Astrup, Kristin Hegstad, Ørjan Samuelsen, Hege Enger, Oliver Kacelnik

**Affiliations:** 1 Department of Antibiotic Resistance and Infection Prevention, Norwegian Institute of Public Health, Oslo, Norway; 2 Norwegian National Advisory Unit on Detection of Antimicrobial Resistance, Department of Microbiology and Infection Control, University Hospital of North Norway, Tromsø, Norway; 3 Research Group of Host-Microbe Interactions, Department of Medical Biology, Faculty of Health Sciences, University of Tromsø –The Artic University of Norway, Tromsø, Norway; 4 Microbial Pharmacology and Population Dynamics Research Group, Department of Pharmacy, Faculty of Health Sciences, University of Tromsø –The Artic University of Norway, Tromsø, Norway; 5 Norwegian Reference Laboratory for MRSA, St. Olavs University Hospital, Trondheim, Norway; Universidade de Lisboa Faculdade de Medicina, PORTUGAL

## Abstract

**Introduction:**

Scandinavian countries have traditionally had a low prevalence of resistant organisms, but have in recent years experienced a change in their epidemiology. We aim to describe the epidemiology of carbapenemase-producing organisms (CPOs), vancomycin-resistant enterococci (VRE) and methicillin-resistant *S*. *aureus* (MRSA) in Norway, measure the importance of infections contracted abroad, and assess the morbidity and mortality associated with these resistant bacteria in Norway.

**Methods and materials:**

We used data from the Norwegian surveillance system for communicable diseases covering all findings of the selected resistant bacteria including both infections and colonisation, in the period 2006–2017. Annual trends were assessed using negative binomial regression. For MRSA, we were able to calculate the Morisita-Horn index and transmission numbers following importation in order to assess the effect this had on further domestic transmission.

**Results:**

The incidence rates (per 100,000 personyears) of the three groups of resistant bacteria have increased during the period. In 2017 the incidence rates were 0.82 for CPOs, 7.09 for VRE and 43.8 for MRSA. 81% of CPO cases were diagnosed in hospitals, but 73% were infected abroad. Most VRE cases were infected in Norwegian hospitals, 85% were associated with hospitals outbreaks. MRSA was predominantly diagnosed in the community, only 21% were diagnosed in hospitals. Of all MRSA cases, 35% were infected in other countries. Most MRSA *spa-*types were not identified again after introduction, resulting in a transmission of MRSA equivalent to a mean of 0.30 persons infected from each *spa-*type identified (range: 0–22). The proportion of infections among all notified cases within each diagnose was 44% for MRSA, 9% for VRE and 45% for CPOs. Among persons notified with bacteraemia, the 30 days all-cause mortality were 20%, 16% and 50% for MRSA, VRE and CPOs respectively.

**Discussion:**

The incidence rates of CPOs, VRE and MRSA in Norway are low, but increasing. The continuing increase of notified resistant bacteria highlights the need for a revision of existing infection prevention and control guidelines.

## Introduction

Antimicrobial resistance has been a challenge to the treatment of bacterial infections across the globe for as long as we have used antibiotics. In recent years, resistance in some pathogenic bacteria that are major causes of healthcare associated infections has become an increasing threat to patients in healthcare institutions. It is in these institutions that the most vulnerable patients are to be found and where it is of vital importance to have effective antibiotic options. Of particular concern is the global increase in prevalence of carbapenem-resistant *Enterobacterales*, *Pseudomonas aeruginosa* and *Acinetobacter baumannii*, vancomycin-resistant enterococci (VRE) and methicillin-resistant *Staphylococcus aureus* (MRSA), all of which are resistant to antibiotics defined as critically important for human medicine [1].

Historically, surveillance data has indicated that Norway, along with other Scandinavian countries, has a low background prevalence of resistant organisms [2–4]. In recent years, however, it appears that the epidemiology has been changing [5–7]. Importantly, the incidence of these different bug-drug combinations may be changing differently; at different rates, in different populations and with differing effect. To counteract the dissemination of multi-drug resistant organisms (MDROs), Scandinavian guidelines advocate the implementation of infection control measures such as screening patients hospitalized abroad and targeted isolation on admission to healthcare institutions [8]. With a changing epidemiology, the question arises of whether these policies will still be effective in the years to come and whether the Scandinavian countries will be able to maintain the current low prevalence of these organisms or if we will see the same rates as those now being found in many other countries in Europe, Asia and the Americas in the future [9].

The aim of this study is to describe the epidemiology of carbapenemase-producing organisms (CPOs), VRE and MRSA in Norway, from 2006 up to and including 2017; and measure the effect of importation of MDROs on the autochthonous epidemiology of a low-prevalence country. Further, we aim to assess the morbidity and mortality associated with these MDROs as a means of describing their impact on public health.

## Materials and methods

### Register

We included data on all human cases (clinical infections and colonization) of CPOs, VRE and MRSA from the Norwegian Surveillance System for Communicable Diseases (MSIS) covering the period 2006–2017. In Norway, all first-time findings of CPO, VRE and MRSA are notifiable to MSIS and the register contains information on both the person (submitted by the treating physician) and the isolate (from the primary laboratory, the reference laboratory, or both), linked by the patient’s unique personal identification (ID) number [10]. Persons with more than one type of notifiable bacteria-resistance combination were treated as one notification per bacteria-resistance type in the analysis. Case data in MSIS includes the patients sex, age, admission to a healthcare institution, travel history, clinical picture, date of death (where applicable), and specific information for each bacterial isolate, including the date the sample was taken, species, clones, and resistance mechanism. Mortality data registered in MSIS is regularly updated from the Norwegian National Population Registry. In this study we had access to mortality data from the period 2006 up to and including 2016.

The use of data from MSIS is regulated by the MSIS-regulation §4.4. The use of data for this work was approved by the Norwegian Institute of Public Health (ref.: 17/11726). The data were analyzed anonymously.

### Population under surveillance

Due to the comprehensive coverage of the MSIS register, there is no selection in this study. We defined the study population as all people in Norway during the study period of 2006 until the end of 2017. However, CPOs were only included in the list of notifiable conditions from 2012. In order to calculate the mortality rate we only included those registered prior to 2017 with a complete personal ID number (implying that they were residents in Norway) in these analyses. For the 30-day and one-year mortality calculation we included persons with a sample taken at least 30 days or 12 months before the end of 2016, respectively.

### Case definitions in MSIS

Carbapenemase-producing organisms (CPOs) are defined as all clinical and screening isolates of *Enterobacterales* (CPEs), *P*. *aeruginosa* or *Acinetobacter* spp. with reduced susceptibility or resistance to meropenem and verified as carbapenemase-producing by the Norwegian National Advisory Unit of Antimicrobial Resistance. Vancomycin-resistant enterococci (VRE) are defined as all enterococci expressing either the *vanA* or *vanB* gene and/or with a minimum inhibitory concentration (MIC) for vancomycin of ≥ 4 mg/l. Methicillin-resistant *Staphylococcus aureus* (MRSA) are defined as all cases of *S*. *aureus* found to be resistant against cefoxitin and expressing either the *mecA* or *mecC* gene and verified as MRSA by the Norwegian Reference Laboratory for MRSA.

### Study-specific definitions

Classification of either infection or colonization at the time of notification was primarily based on the diagnosis given by the reporting physician. We supplemented this information using the material of the specimen and clinical information in the reporting form. Based on this information the classification of severe infections included pneumonia, bloodstream infection, meningitis, encephalitis and necrotising fasciitis. We used the name of the notifying ward together with information of place of treatment given in the free-text fields, to identify patients diagnosed in hospitals, including intensive care units (ICUs). Imported cases were cases where the treating physician had reported the MDRO as acquired in another country. When the country of acquisition was not specified, we defined the case as imported when the information given in free-text fields indicated acquisition abroad. For the purpose of this study, we defined livestock associated MRSA (LA-MRSA) in two groups: all isolates of clonal complex (CC) 398; and all isolates Panton-Valentine Leukocidin (PVL) negative and from *Staphylococcus* protein A (*spa*) types previously associated with outbreaks in Norwegian livestock, as described by Elstrøm et al. [11].

### Statistical analyses

Descriptive and analytical statistics were performed using Stata v15, Stata Corp. When assessing the annual trend of notified cases, we used negative binomial regression after organizing the data as yearly time-series data. The annual number of cases were tested and we found no significant autocorrelation [12]. We used separate regression models for the yearly trend for each diagnosis, each place of acquisition and each place of diagnosis, and with the yearly number of cases as numerator and the annual total population in Norway as the denominator (Table C in S1 File). To assess the effect importation has on the epidemiology of MRSA in Norway, we used the Morisita-Horn index as a measure of the abundance of overlapping MRSA *spa-*types in imported and domestic cases. We calculated the Morisita-Horn index (MH) using the formula

$$MH=\frac{2\sum_{i=1}^{S}x_{i}y_{i}}{\left(\frac{\sum_{i=1}^{s}x_{i}^{2}}{X^{2}}+\frac{\sum_{i=1}^{s}y_{i}^{2}}{Y^{2}}\right)XY}$$

where *x*_*i*_ is the number of times *spa-*type *i* is represented in the total of imported cases (*X*), *y*_*i*_ is the number of times *spa-*type *i* is represented in the total of domestic cases (*Y*), and *S* is the number of unique *spa-*types reported. Further, we identified unique *spa-*types found after 2007 which were not notified in the two first years of the study period, and where the first notified case was reported as infected abroad. When the *spa*-type was not present in the two first years of the study period, we treated it as a new *spa*-type in Norway. We followed these newly imported *spa-*types over the next 10 years and calculated a transmission number after introduction to Norway, as the number of persons infected in Norway by each *spa-*type per person importing the *spa-*type to Norway. The transmission number (TN) was calculated using the formula

$$TN=\frac{y_{i}}{x_{i}}$$

where *y*_*i*_ is the number of new domestic cases of the *spa-*type *i* notified after the first case were imported, and *x*_*i*_ is the number of imported cases of the *spa-*type *i* notified after 2007.

## Results

### Description of the epidemiology

**Fig 1:**
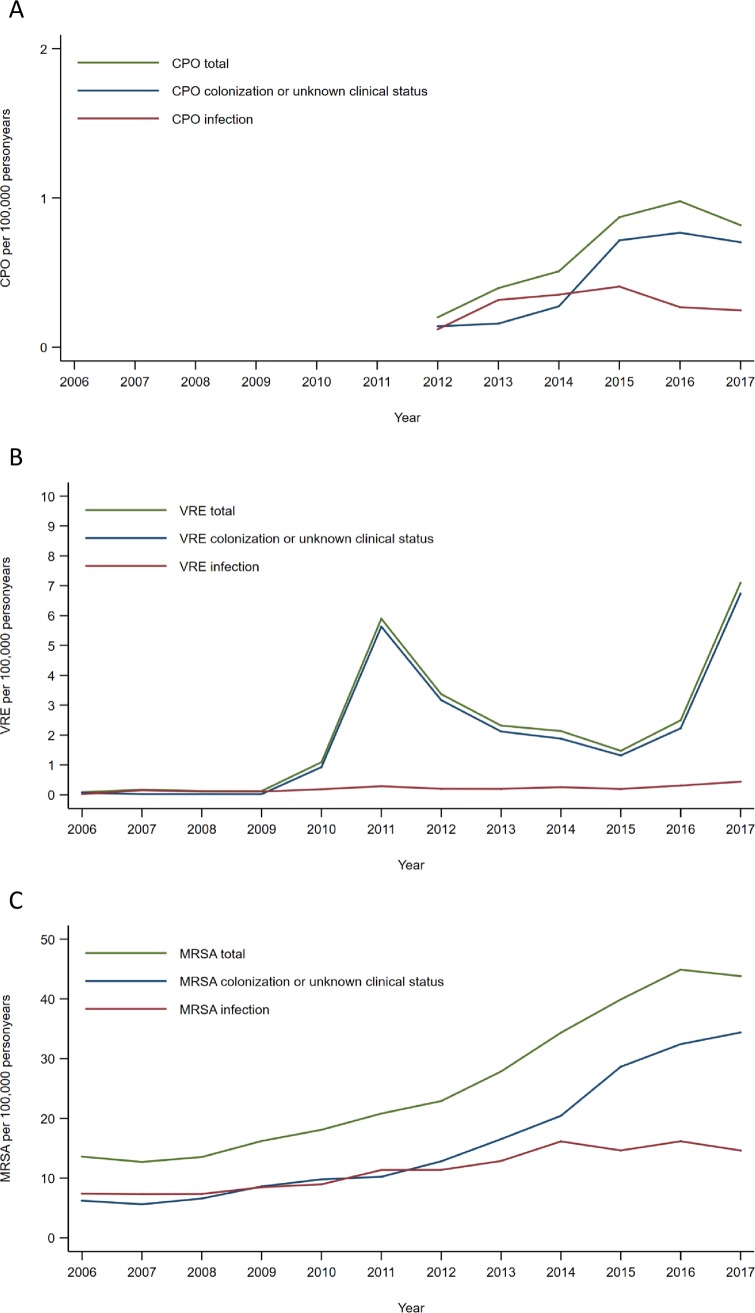
**Incidence rate (number of persons notified per 100,000 personyears) of CPO (A), VRE (B) and MRSA (C).** Note the different scales on the y-axis.

From 2006 up to and including 2017, a total of 18,155 notifications in 17,082 persons with CPOs, VRE or MRSA, were registered in MSIS. Of these, 15,547 people were notified with MRSA. The incidence rates (number of persons per 100,000 personyears (IR)) in 2017 were 0.82 for CPOs, 7.09 for VRE and 43.80 for MRSA. During the study period there has been a significant increase in the annual number of persons registered within each group (bug-drug combination and infection/colonization), except for persons notified with CPO infections (Fig 1 and Table A in S1 File). However, in the last four years the incidence rate of MRSA infections has plateaued, reflected in an incidence rate ratio (IRR) for MRSA between 2014 and 2017 of 0.98 (95% CI 0.94–1.02).

A majority of the people notified with VRE or CPOs were males (≥60%) and of high median age (>60 years) (Table 1). MRSA notifications were evenly distributed between men and women. The median age was 32 with an interquartile range (IQR) of 33 years. For all three groups of MDROs the full age-range was from one year or below, to over 95 (Table 1 and Fig A in S1 File).

**Table 1:** Number of cases and distribution of sex, age and place of diagnosis. Note that the values represent absolute numbers and (percentages) for each diagnosis.

	CPO	VRE	MRSA
No. of notifications	231	1,350	16,574
No. of persons	195	1,340	15,547
Males (%)	126 (65)	798 (60)	7,706 (50)
Mean age (min-max)	57 (1–96)	68 (0–97)	36 (0–104)
Median age (p25-p75)	62 (42–72)	72 (59–81)	32 (19–52)
Diagnosed by general practitioners (%)	25 (13)	57 (4)	11,471 (74)
Diagnosed in hospitals (%)	168 (86)	1,261 (94)	3,282 (21)
Diagnosed in intensive care units (ICUs) (%)	21 (11)	30 (2)	120 (1)
Diagnosed in long-term care facilities (%)	2 (1)	22 (2)	794 (5)
Registered as healthcare workers (%)	2 (1)	1 (0)	737 (5)

**Fig 2:**
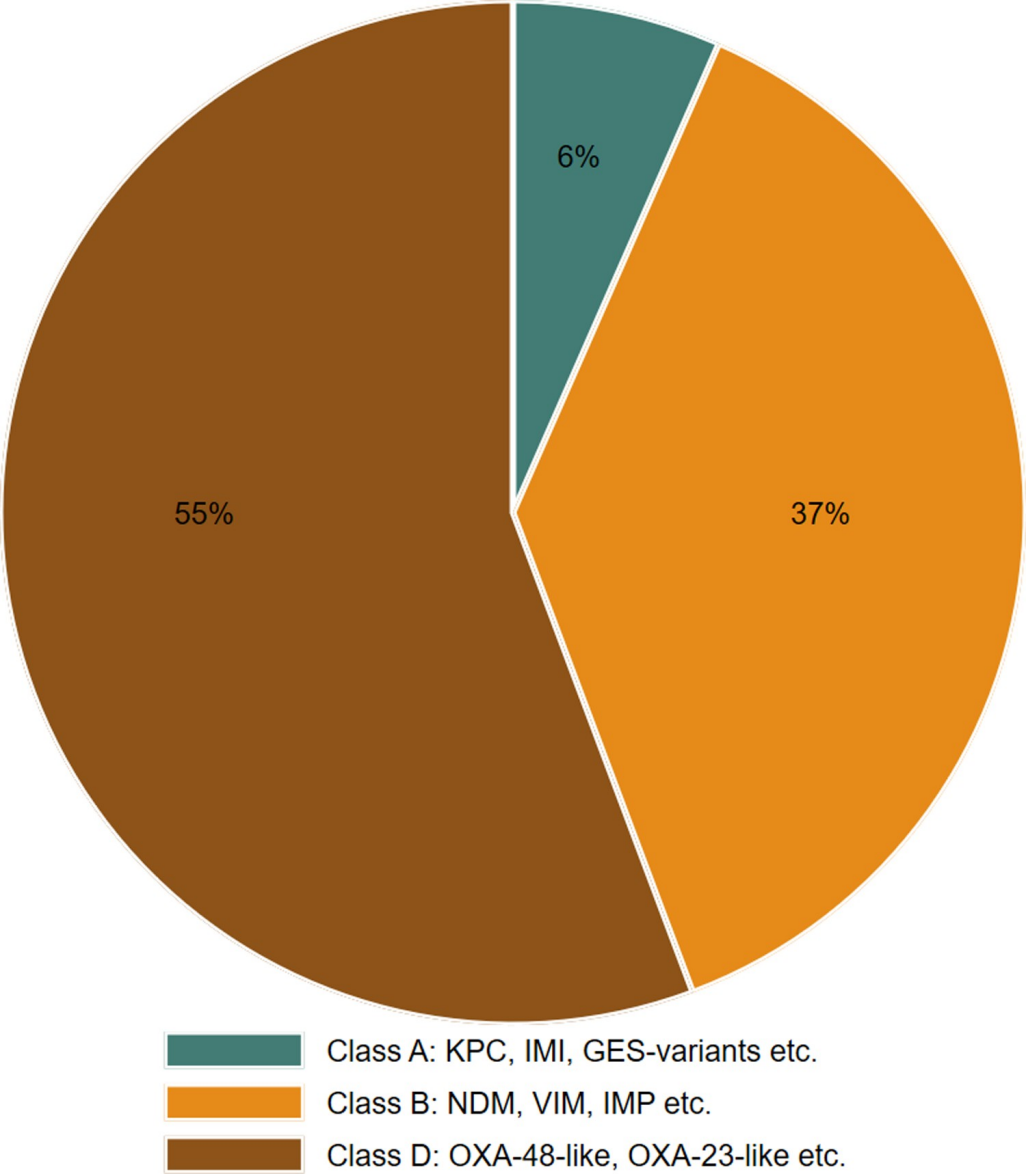
CPOs distributed according to the Ambler classification.

Notification of CPOs was introduced in 2012, and the total number in the study period was 231 notifications from 195 persons. These included CPEs (n = 137), *Acinetobacter spp*. (n = 69) and *Pseudomonas spp*. (n = 25). Almost all cases (96%) of carbapenemase-producing *Acinetobacter* and *Pseudomonas*, and 81% of all CPEs were diagnosed in hospitals. Based on the Ambler classification, the vast majority of carbapenemases belonged to class B or D (Fig 2). The New Delhi metallo-β-lactamase (NDM) (n = 59), OXA-48-like (n = 57) and OXA-23-like (n = 50) were the dominating carbapenemase variants identified, and the number of isolates with these types of resistance mechanisms increased during the study period.

**Fig 3:**
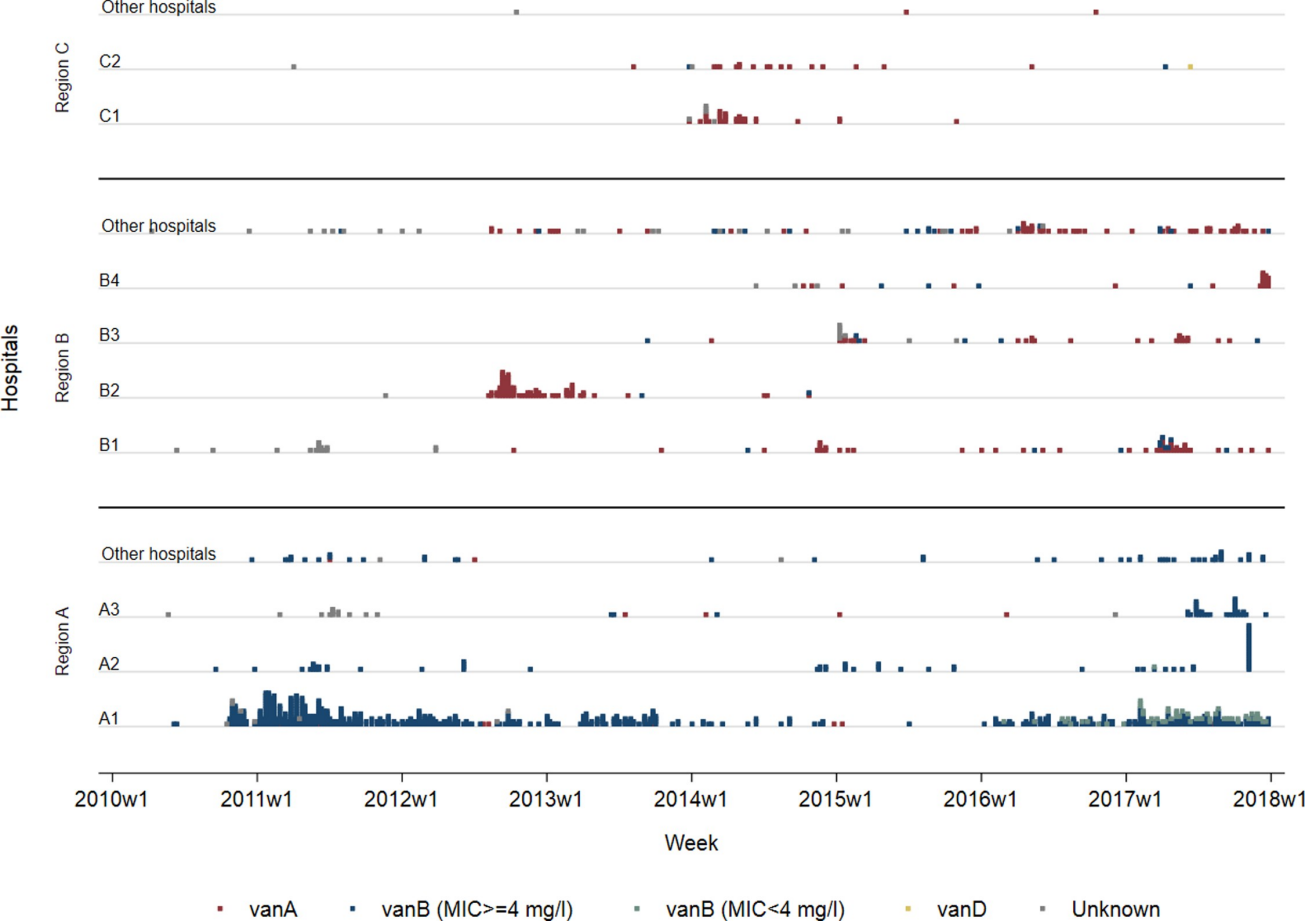
Number of persons registered with VRE in hospitals per week from week 1 in 2010 and up to 2018 (2010w1 – 2018w1), distributed by hospitals and genes for vancomycin resistance. The hospitals have been grouped in the three Health trust regions with registered VRE-outbreaks in the period, and hospitals in each region with less than 20 cases have been aggregated together. Each coloured box represent one person.

The specific *Enterococcus* species (*faecium*, *faecalis* etc.) has only been registered in MSIS since the end of 2015. Thus, we can only describe species-specific VRE-data from the last two years, where 476 persons with *E*. *faecium* were reported compared to eleven cases of *E*. *faecalis*. In total 1,261 (94%) of the VRE cases were diagnosed in hospitals and over 85% of all persons notified were associated with recognised hospital outbreaks. The majority of positive isolates were found in asymptomatic carriers during case tracing. The first multi-ward outbreak of VRE in Norway occurred in 2010, with an increasing number of hospital outbreaks every year apart from 2016 (Fig 3). During the study period there was only one VRE-outbreak in a long-term care facility (reported in 2017) and in total 22 persons were diagnosed with VRE during their stay in long-term care institutions, including six residents linked to the reported outbreak. The first registered VRE-outbreak was a *vanB* associated outbreak at one hospital (hospital A1) which altogether notified 74% of all cases identified in the study period harbouring the *vanB* gene (Fig 3). This resulted in more cases of *vanB* than *vanA* at the national level even though *vanA* has been involved in more outbreaks.

MRSA was predominantly diagnosed by general practitioners, only 3,282 (21%) of the MRSA cases were diagnosed in hospitals and 794 (5%) were diagnosed during their stay in long-term care institutions. In Norway, there are restrictions regarding patient related work for MRSA positive healthcare workers, and thus they are screened before employment if MRSA exposure is reported. In total, 737 (5%) of all persons notified with MRSA were reported to be healthcare workers. Among all MRSA notifications, 779 (5%) could be linked to known outbreaks of MRSA in hospitals (n = 40 outbreaks), in long-term care facilities (n = 92 outbreaks) or in different settings in the community (n = 10 outbreaks).

Sequencing of the repeat region of the *spa-*gene in order to genotype the isolates was performed on isolates from 14,529 (93%) persons notified with MRSA. These isolates belonged to 1,092 different *spa*-types, where 805 (74%) were found less than 5 times during the study period (Fig B in S1 File). The five *spa-*types most frequently reported were t002 (n = 1,382), t019 (n = 1,081), t008 (n = 1,003), t223 (n = 882) and t127 (n = 664). Norway has implemented a national strategy against LA-MRSA [11, 13]. In the study period, we found MRSA *spa-*types belonging to the clonal complex (CC) 398 in 185 persons notified to MSIS. Of these, 118 were PVL negative and belonged to MRSA strains often associated with livestock in Europe [14]. A few outbreaks of MRSA in Norwegian livestock have been discovered yearly since 2014. In total 52 persons diagnosed with MRSA could be directly linked to known MRSA positive Norwegian pig herds (Table B in S1 File). So far, all LA-MRSA outbreaks have been successfully controlled [11, 13].

### Imported versus domestic cases

We found a significant increase of notifications during the period in both persons infected abroad and in those infected in Norway for all three groups of MDROs, except for persons infected with VRE in Norway (Table 2). Countries in Asia were the most often reported countries of acquisition, followed by countries in the southern and eastern part of Europe.

**Table 2:** Results of negative binomial regression of the annual number of cases, 2006–2017, distributed by diagnosis, place of acquisition and place of diagnosis. LA-MRSA included persons notified with PVL-negative isolates belonging to CC398 or CC1 and the same spa-types found in known outbreaks in Norwegian swineherds (spa-types t011, t034, t12359, t177).

DIAGNOSIS	IRR (Ratio of mean annual cases)	95% CI	p-value
***CPO***			
Persons infected in other countries	1.25	1.11–1.41	<0.05
Persons infected in Norway	1.31	1.04–1.66	<0.05
***VRE***			
Persons infected in other countries	1.54	1.38–1.71	<0.05
Persons infected in Norway	1.32	0.99–1.74	0.06
***MRSA***			
Persons infected in other countries	1.18	1.15–1.20	<0.05
Persons infected in Norway	1.06	1.04–1.07	<0.05
***MRSA diagnosed by general practitioners***			
Total number of persons notified	1.17	1.15–1.18	<0.05
Persons infected in other countries	1.19	1.16–1.22	<0.05
Persons infected in Norway	1.09	1.07–1.11	<0.05
***MRSA diagnosed in hospitals***			
Total number of persons notified	1.12	1.10–1.14	<0.05
Persons infected in other countries	1.14	1.10–1.17	<0.05
Persons infected in Norway	1.03	1.01–1.05	<0.05
***LA-MRSA associated with outbreaks in livestock in Norway***			
Total number of persons notified	1.53	1.26–1.86	<0.05
Persons infected in other countries	1.31	1.14–1.49	<0.05
Persons infected in Norway	1.47	1.12–1.93	<0.05

Only a few cases of VRE (6%) were reported to be acquired abroad, while most CPO cases (73%) were reported infected outside Norway. A total of 52 persons notified with CPOs were not reported to be acquired abroad, equally distributed between infected in Norway and unknown place of infection. Among the persons not reported infected abroad, 23 (44%) were diagnosed with CPE, mainly *E*. *coli* or *K*. *pneumoniae*, producing the OXA-48-like carbapenemase. The total number of notifications of OXA-48-like CPOs (both domestic and imported cases) has increased from three in 2006 to 24 in 2017. Based on the reported information, none of the domestic cases or cases with unknown place of infection formed clusters of persons living in the same municipalities or with known epidemiological links.

**Fig 4:**
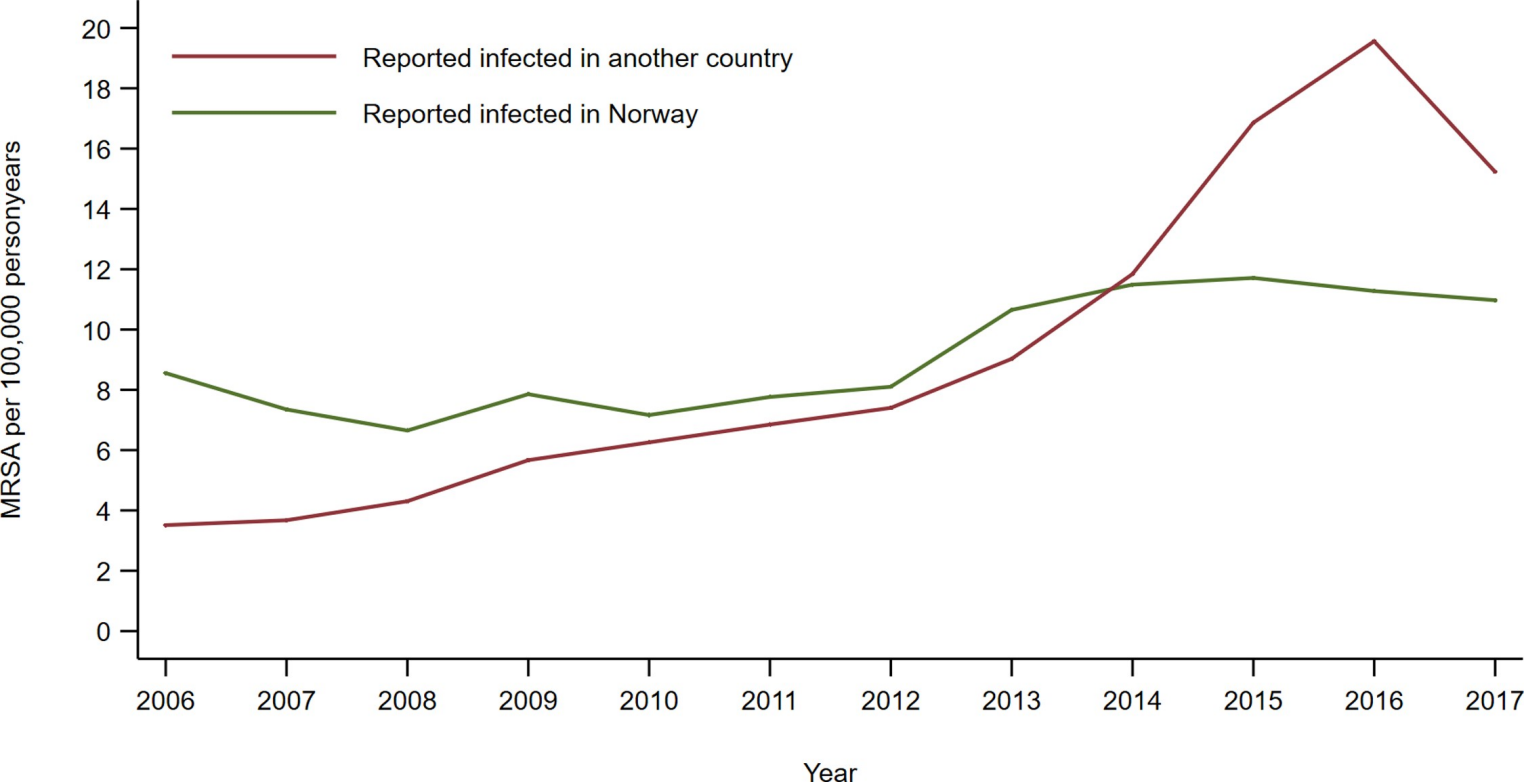
Incidence rate (number of persons notified per 100,000 personyears) of MRSA, by place of acquisition.

Persons diagnosed with MRSA were divided between 35% infected in Norway and 36% infected in other countries, and the rest (29%) were reported with no information on place of acquisition. The increase in both imported and domestic MRSA cases was significant both among patients in hospitals, in long-term care facilities or diagnosed by their general practitioners (Table 2). Although we see a decrease in imported MRSA cases in 2017, there was still a steep rise of persons infected abroad over the whole study period (Table 2 and Fig 4).

**Fig 5:**
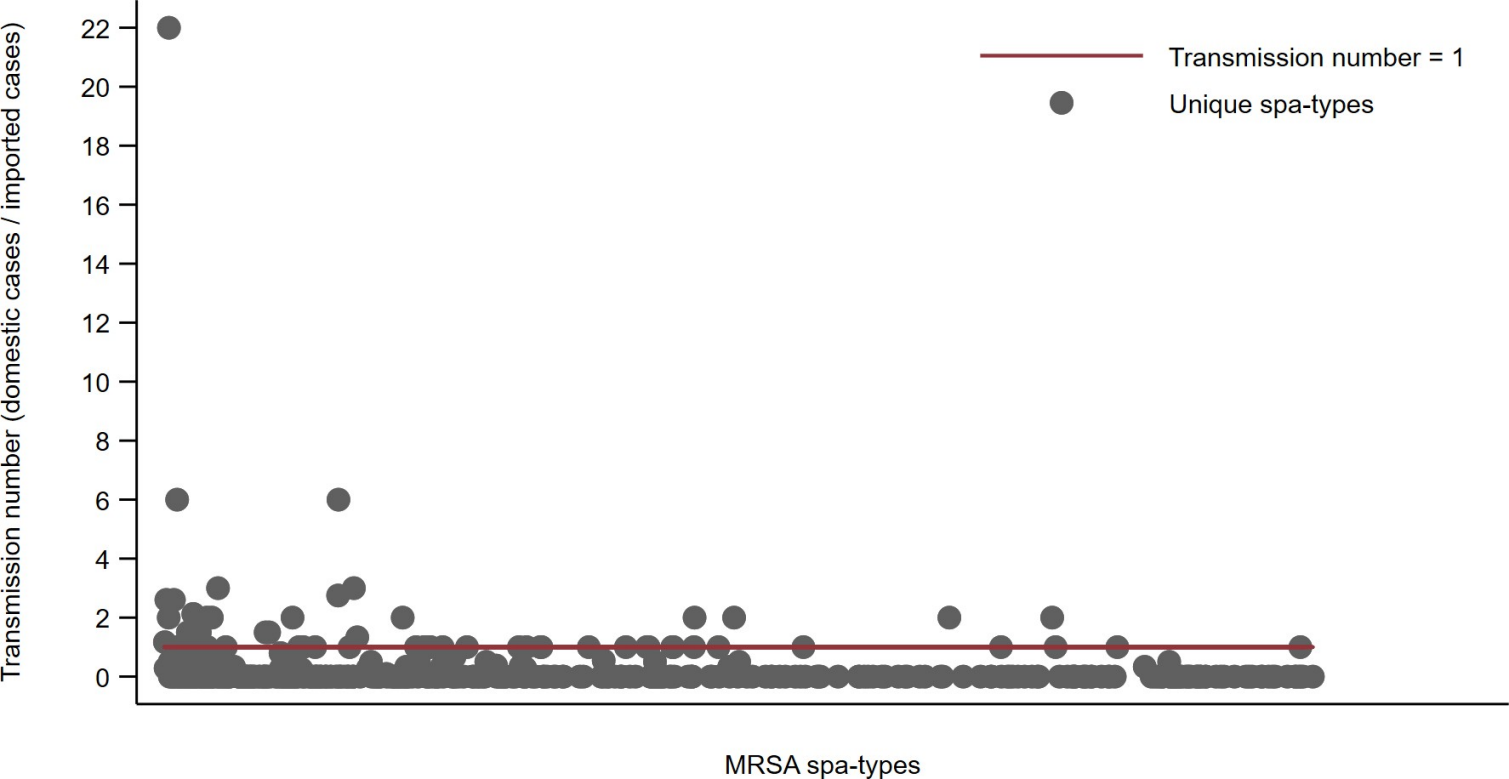
Transmission number (domestic cases/imported cases) per unique MRSA *spa-*type not previously identified and primarily diagnosed in persons infected abroad, in the period 2008–2017.

There was a high diversity of MRSA *spa-*types in both imported and domestic cases. The Morisita-Horn’s overlap index (a measure of similarity) for domestic and imported cases was close to zero throughout the study period (Fig C in S1 File). During the first two years, 151 different *spa-*types were identified. In the following ten years a total of 941 new *spa*-types were found. Among these, 426 (45%) of the new *spa*-types were primarily identified in persons infected abroad while 236 (25%) were primarily identified in persons infected in Norway (Fig D in S1 File). A total of 324 of 426 (76%) of new *spa*-types diagnosed in persons infected abroad were not later identified among persons infected in Norway. The mean transmission number (TN) for all new imported *spa*-types from 2008 to 2017, calculated as the number of persons infected in Norway per persons infected abroad per unique spa-type, was 0.30 and ranged from 0 to 22 (Fig 5).

### Morbidity and mortality

During the study period, 41% of the notifications were clinical infections. The proportion of infections was highest among patients diagnosed with CPO or MRSA (45 and 44% respectively), while only nine percent of VRE cases were notified as infections. However, VRE had the highest proportion of reported bacteraemia amongst those notified with infection (30%), whilst 16% of all CPO infections and only 2% of those notified with MRSA infections were bacteraemia. 30-day mortality among patients notified with bacteraemia was highest in those with CPO infections (50%) followed by MRSA (20%) and VRE (16%). Table 3 shows the morbidity and mortality outcomes for the different subpopulations in this study.

**Table 3:** Number of persons notified with infections or reported dead within 30 days or 1 year after date of diagnosis. Mortality is calculated for persons with a complete national identification number and diagnosed with a MDRO 1 year or 30 days prior to death. Severe infections identified in MSIS included pneumonia, bloodstream infections, meningitis, encephalitis and necrotising fasciitis.

	CPO	VRE	MRSA
Infections (% of all notified cases)	88 (45)	123 (9)	6,833 (44)
Severe infections (% of cases with infections)	20 (23)	38 (31)	217 (3)
Bacteraemia (% of cases with infections)	14 (16)	37 (30)	140 (2)
1 year all-cause mortality (% of all notified cases)	36 (18)	336 (25)	746 (5)
30 days all-cause mortality (% of all notified cases)	22 (11)	113 (8)	178 (1)
30 days all-cause mortality in hospitalized patients (% of cases diagnosed in hospitals)	22 (13)	111 (9)	117 (4)
30 days all-cause mortality in patients with infections (% of cases with infections)	16 (18)	14 (11)	106 (2)
30 days all-cause mortality in patients with notified bacteraemia (% of cases with bacteraemia)	7 (50)	6 (16)	28 (20)

## Discussion

Compared to most other countries, Norway still has a low incidence rate of CPOs, VRE and MRSA [2, 15]. However, our data show an increasing trend of notifications for all of these MDROs, and the increase is ongoing despite the development and implementation of comprehensive infection prevention and control measures, including antibiotic stewardship, in Norwegian health care [16].

Several countries which today are considered to have a high endemic level of MDROs in hospitals, had comparable rates in the recent past to those seen in Scandinavian countries today [17]. Based on the present epidemiological situation in low-endemic countries, it is opportune to ask if the control regimes implemented against resistant bacteria will continue to be effective, or if new approaches are needed. The different Scandinavian guidelines against MDROs all advise several bundles of measures in hospitals and long-term care institutions, while control of transmission in the community is less established [8]. In addition, many of the measures advised in these guidelines lack evidence for their effectiveness [18–21]. The Scandinavian countries may have reached a crossroads where we either continue to follow the current prevention and control measures and trust that they are maintaining a low level of severe MDRO infections, or we reinforce the current practice with both an evaluation of the routines in healthcare institutions together with measures that prevent and control the spread of CPOs, VRE and MRSA in the community. The latter may be needed in order to prevent an increasing infection pressure and influx from the community into hospitals and long-term care institutions. However, the analysis in this study makes it clear that different factors influence the notification rate of each type of MDRO, and a common approach to prevent the spread of CPOs, VRE and MRSA in different settings and populations may not be optimal for each pathogen.

The epidemiology of VRE in Norway has so far mainly been a local problem driven by outbreaks in a few hospitals. Notified cases in the last two years have been dominated by *Enterococcus faecium*. Several studies from other countries have identified a shift in the distribution of species from *E*. *faecalis* to more resistant clones of *E*. *faecium* [22, 23]. This shift is explained by an increased selection and spread of hospital adapted clones of *E*. *faecium* [24–26]. One reason why we may have seen a rise in the prevalence of VRE in our hospitals, is the previously mentioned adaptation of *E*. *faecium* to our hospital environment. This adaptation is probably due to the development of resistance to different biocides, including alcohols, and antibiotics along with an ability to survive on dry surfaces for up to several years [27, 28]. Most cases in Norway are also identified with the *vanB* resistance gene cluster and only a few cases of VRE seem to be due to infection abroad. The onset of an outbreak with *vanB* VRE often occurs due to transfer of the *vanB* gene cluster from the normal gut flora in a patient during antibiotic treatment [29]. In addition *vanB* VRE might be more difficult to detect phenotypically because of low level vancomycin resistance, which increase the risk of spread in the hospital setting before outbreaks are discovered. As seen in Fig 3, in the last few years we have identified an increase of *vanB* VRE with a MIC below 4 mg/l. The dissemination of low level resistant VRE might be one of the explanations why we have had a large ongoing VRE outbreak in a hospital in Norway and several *vanB* VRE outbreaks in other Scandinavian hospitals. The solutions to this challenge, presented in international studies, are prudent antibiotic use and both standard and contact precautions [18, 30, 31]. However, their relevance for low prevalence settings remains unclear. An important question is how the strong effort in VRE prevention and control already in place in many Scandinavian hospitals can be further improved.

Contrary to VRE, CPOs in Norway are currently mainly diagnosed in patients directly transferred from foreign hospitals or with a history of hospitalization in other countries [7, 32, 33]. So far, only a few cases were assessed as infected domestically. This highlights the importance of screening for MDROs, including CPOs, especially when patients have been treated in hospitals abroad. However, the epidemiology described here can also be an artefact of the surveillance system itself. The guidelines and awareness of CPOs may not be sensitive enough to identify carriers that have not been in a hospital abroad, but have been infected in other ways. The Norwegian surveillance system of resistance in microbes (NORM) agrees that the prevalence of CPOs is still low in Norway, but also reveals a steady increase of extended-spectrum β-lactamase (ESBL) production in the selected clinical isolates of *E*. *coli* and *K*. *pneumoniae* collected during the last decade [34]. Scandinavian countries might be in a unique situation with a low prevalence of CPOs, but we must expect it to increase rapidly and in the same way as seen in many European countries, unless current guidelines are continuously evaluated, updated and adhered to. Further, we need to define cost-appropriate measures that can be applied in this setting.

Detailed and comprehensive MRSA guidelines are well implemented in Norwegian hospitals and the results in our study indicate that both hospitals and long-term care institutions to a large extent manage to control the transmission of MRSA between inpatients. However, it seems that one of the main factors influencing the incidence rate is increasing importation of MRSA from other countries. Most notified cases are diagnosed in the community where few measures are in place to prevent further transmission.

MRSA colonization is mainly found through targeted screening and the number of MRSA cases reported in this study may suffer from detection bias. A revision of the national MRSA guidelines was published in 2009 and implemented gradually in the different health regions over the following years. The increase in the number of notified persons with MRSA has probably been influenced by a gradually more active screening policy. However, the indication for taking clinical samples has not changed and the annual IR of MRSA infections has also increased. This would support the finding of an overall increase in the incidence of MRSA in Norway. Over the last four years, the rate of increase in infections has tailed off compared to notifications of colonizations, which may mean that we are reaching a steady state for MRSA in the country.

The number of people that one infected person will infect (the reproduction number) may vary between the MDROs, and it is influenced by the settings (i.e. hospitals versus community) and by the population at risk affected by immunity status, invasive medical treatment etc. The definition of the reproduction numbers for MDROs is made even more difficult to assess because of the asymptomatic carrier state. In this study, we used the data available in MSIS to measure the distribution of imported and domestic MRSA cases together with genotypes of isolates to assess the cumulative number of new domestic cases after importation. The low Morisita-Horn index together with a low transmission number for most newly imported *spa-*types indicates that the reproduction number of MRSA in the community and in healthcare institutions is often below 1, which means that further spread within each setting should stop [35]. This is comparable to the reproduction number assessed per hospital stay for MRSA positive inpatients in the Netherlands [36]. However, we also found an annual increase in the IR during the study period. Our assessment of the transmission number was based on MRSA *spa-*types imported for the first time after 2007, and the five most common notified MRSA *spa-*types in Norway were already established in 2006/2007 and thus not included in the calculation of the transmission number. This may have contributed to an underestimation of the potential for transmission of MRSA. Even though most imported MRSA strains after 2007 seem not to have led to significant domestic spread, our results show that the potential for transmission varies a lot between the strains. Although we experience that many MDROs imported to Scandinavian countries do not lead to further domestic spread, we also identify continuing transmission of single strains in the community and outbreaks in healthcare institutions [5].

The Norwegian government have launched a national AMR strategy where the main objective is to decrease the antibiotic use by 30%. The work on antibiotic stewardship is important to prevent the development and selection of antibiotic resistant bacteria. However, even If we manage to reach this target, the epidemiology of MDROs will still be largely influenced by importation, especially when it comes to CPOs and MRSA. Thus, other measures to prevent and control multi-resistant bacteria, including screening and standard precautions, are also important for reducing the growth of AMR in Norwegian healthcare institutions.

At present, it seems that the resistance in the Gram-positive bacteria investigated in this study had less impact on disease outcome compared to what is reported in most other international studies [37–41]. Although all cases of selected MDROs diagnosed in Norway are reported to MSIS, the data in the surveillance system suffer from detection bias which may both overestimate and underestimate the assessed morbidity and mortality. A large proportion of the persons notified with CPOs seems to be elderly patients with a statistically higher risk of infection. Many of the patients notified with CPOs have been directly transferred from foreign hospitals, which may indicate severe trauma or diseases demanding comprehensive and invasive treatment. Thus, the high risk of mortality after CPO bacteraemia, may not just be due to lack of first-line antibiotic treatment but may also be influenced by the generally high vulnerability in this group of patients. The risk of mortality after VRE bacteraemia is lower than assessed in several international studies [42, 43]. However, some studies have shown that the risk of mortality attributable to *vanB* VRE (responsible for the largest single outbreak) is not increased compared to antibiotic susceptible *Enterococcus* [44]. In notified cases of MRSA bacteraemia we find a mortality within 30 days comparable to what other Norwegian studies have found in methicillin sensitive *S*. *aureus* (MSSA) [45, 46]. A lower percentage of severe infections among both VRE and MRSA cases, may partly be due to our screening and case-finding policies. The notified cases of these two diseases have a higher amount of carriers than what is reported for persons with CPOs. The active surveillance in Norway with comprehensive search for MRSA and VRE may contribute to a known carrier status in patients when clinical signs of infections occur, and thus increases the possibility to choose an empirical treatment the bacteria are susceptible to. If this is a factor influencing the low risk of mortality attributable to MRSA or VRE in countries with active surveillance, it will be an important positive effect of a comprehensive search strategy. However, with increasing domestic spread of MDROs, it will be more difficult to perform a targeted screening that effectively detect most asymptomatic carriers. This will lead to more introduction and spread of MDROs in hospitals and increased risk of infections and deaths comparable to what is reported from countries outside Scandinavia.

It is difficult to predict the future MDRO situation in the Scandinavian countries, but without increasing awareness and implementation of effective prevention measures, we will probably not be able to decelerate the importation and spread of MDROs. The continuing increase of notified cases over the last 12 years, highlights the need of revision of the existing infection prevention and control. This applies both to an evaluation of the standard precautions and the MDRO specific control measures implemented in health care. The Norwegian government has this year started the development of a national action plan for infection prevention and control. The authors hope this will contribute to keep these MDROs under control. However, we lack high quality studies assessing the effect of MDRO prevention and control measures in low endemic settings, and we need to strengthen the capacity and possibility to perform such studies in the Scandinavian countries.

## Supporting information

S1 File Table A. Results of negative binomial regression of the changing annual number of cases, 2006–2017.
Figure A. Histogram of people notified with MRSA, VRE or CPO, distributed by five year age groups.
Figure B. Number of persons notified by each MRSA spa-type.
**Table B. Annual number and percentage of persons notified with livestock associated MRSA in Norway.** MRSA CC398 included all persons notified with CC398 (PVL-negative or positive). LA-MRSA in Norway included persons notified with PVL-negative isolates belonging to CC398 or CC1 and the same spa-types found in known outbreaks in Norwegian swineherds (spa-types t011, t034, t12359, t177). LA-MRSA and known livestock contact included persons notified with PVL-negative isolates belonging to CC398 or CC1 and the same spa-types found in known outbreaks in Norwegian swineherds, and working with MRSA positive pigs in Norway or being household member of a person working with MRSA positive pigs in Norway.
**Figure C. The Morisita-Horn’s index per year of overlapping spa-types identified in the two groups: persons infected in Norway and persons infected in another country.** An index close to 0 indicate little or no overlap while an index close to 1 indicate that the same spa-types occur in almost the same proportions in both samples.
Figure D. Number of unique MRSA spa-types per year, by place of acquisition.
Table C. Syntaxes used in Stata v15 for regression models of trends.
(DOCX)
