# Supplementary material for: The fight to keep resistance at bay, epidemiology of carbapenemase producing organisms (CPOs), vancomycin resistant enterococci (VRE) and methicillin resistant Staphylococcus aureus (MRSA) in Norway, 2006 - 2017
Source: PLoS One. 2019 Feb 4;14(2):e0211741. doi: 10.1371/journal.pone.0211741 (PMC6361454; doi:10.1371/journal.pone.0211741)
Supplement: S1 File — Table A. Results of negative binomial regression of the changing annual number of cases, 2006–2017. Figure A. Histogram of people notified with MRSA, VRE or CPO, distributed by five year age groups. Figure B. Number of persons notified by each MRSA spa-type. Table B. Annual number and percentage of persons notified with livestock associated MRSA in Norway. MRSA CC398 included all persons notified with CC398 (PVL-negative or positive). LA-MRSA in Norway included persons notified with PVL-negative isolates belonging to CC398 or CC1 and the same spa-types found in known outbreaks in Norwegian swineherds (spa-types t011, t034, t12359, t177). LA-MRSA and known livestock contact included persons notified with PVL-negative isolates belonging to CC398 or CC1 and the same spa-types found in known outbreaks in Norwegian swineherds, and working with MRSA positive pigs in Norway or being household member of a person working with MRSA positive pigs in Norway. Figure C. The Morisita-Horn’s index per year of overlapping spa-types identified in the two groups: persons infected in Norway and persons infected in another country. An index close to 0 indicate little or no overlap while an index close to 1 indicate that the same spa-types occur in almost the same proportions in both samples. Figure D. Number of unique MRSA spa-types per year, by place of acquisition. Table C. Syntaxes used in Stata v15 for regression models of trends. (DOCX) [file pone.0211741.s001.docx]

## **S1 file. Supporting tables and figures**

**Table A**

| Diagnosis | IRR (Ratio of mean annual cases) | 95% CI | p-value |
| --- | --- | --- | --- |
| ***CPO*** |  |  |  |
| Total number of persons notified | 1.30 | 1.15 – 1.47 | <0.05 |
| Persons notified with infections | 1.06 | 0.91 – 1.23 | 0.466 |
| Persons notified with colonisations or unknown clinical status | 1.61 | 1.32 – 1.98 | <0.05 |
| ***VRE*** |  |  |  |
| Total number of persons notified | 1.40 | 1.14 – 1.72 | <0.05 |
| Persons notified with infections | 1.14 | 1.08 – 1.20 | <0.05 |
| Persons notified with colonisations or unknown clinical status | 1.50 | 1.10 – 2.04 | <0.05 |
| ***MRSA*** |  |  |  |
| Total number of persons notified | 1.14 | 1.12 – 1.15 | <0.05 |
| Persons notified with infections | 1.09 | 1.07 – 1.10 | <0.05 |
| Persons notified with colonisations or unknown clinical status | 1.18 | 1.16 – 1.21 | <0.05 |

**Figure A**
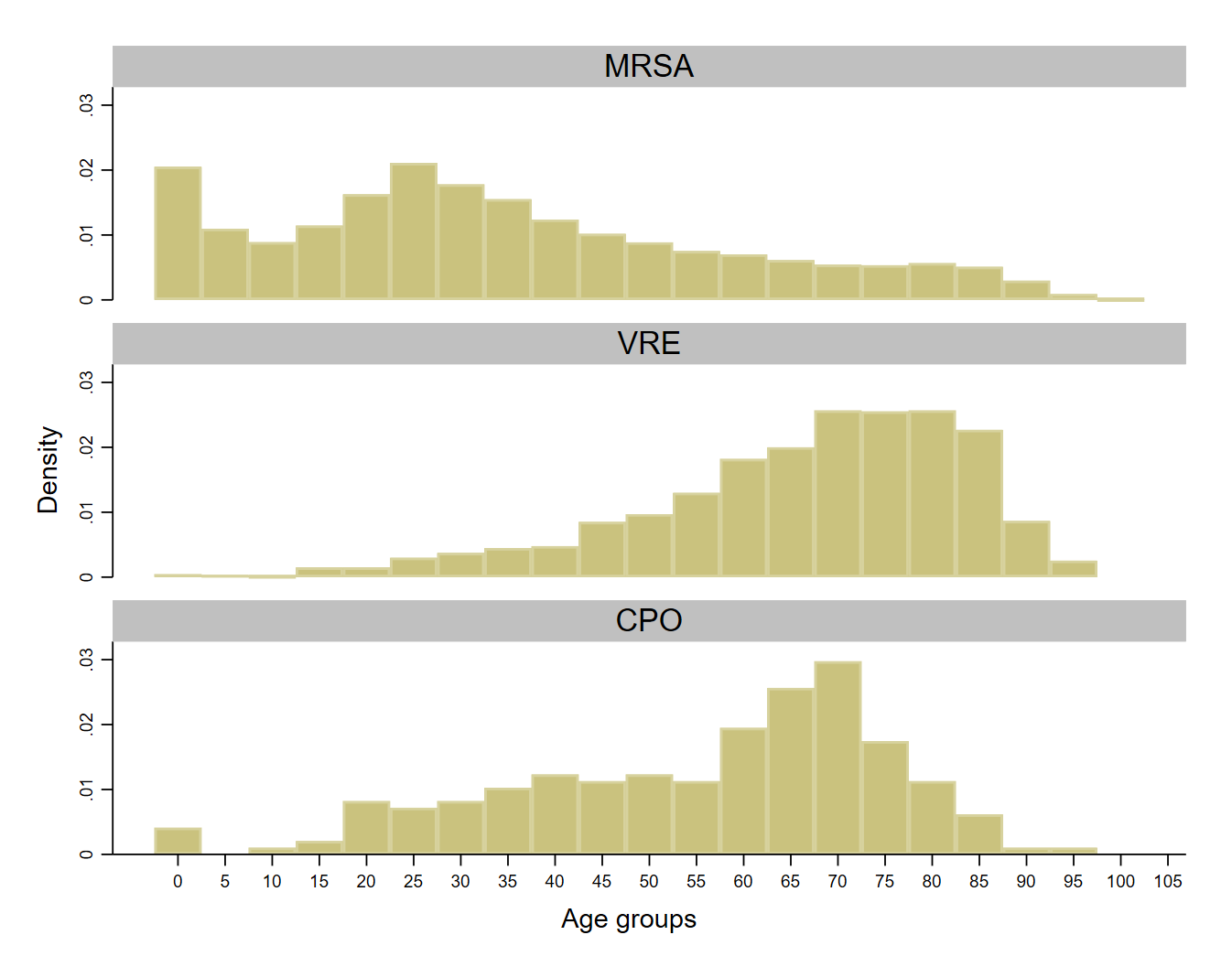


**Figure B**

*
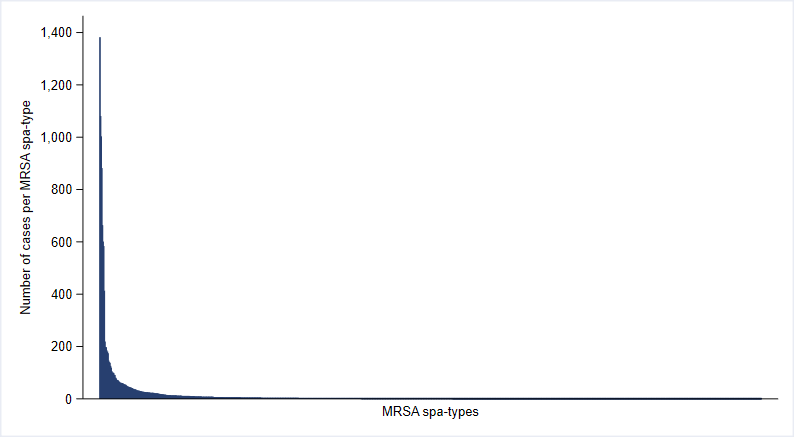
*

**Table B**

| **Year** | **MRSA CC398 (%)** | **LA-MRSA in Norway (%)** | **LA-MRSA and known livestock contact (%)** | **MRSA total, all *spa-*types** |
| --- | --- | --- | --- | --- |
| 2006 | 0 | 0 | 0 | 630 |
| 2007 | 0 | 0 | 0 | 594 |
| 2008 | 0 | 1 (0.16) | 0 | 640 |
| 2009 | 2 (0.26) | 2 (0.26) | 0 | 777 |
| 2010 | 2 (0.23) | 2 (0.23) | 0 | 878 |
| 2011 | 2 (0.20) | 2 (0.20) | 0 | 1,023 |
| 2012 | 7 (0.61) | 4 (0.35) | 0 | 1,141 |
| 2013 | 46 (3.27) | 43 (3.06) | 30 (2.13) | 1,406 |
| 2014 | 24 (1.37) | 15 (0.86) | 6 (0.34) | 1,754 |
| 2015 | 31 (1.50) | 22 (1.07) | 14 (0.68) | 2,061 |
| 2016 | 31 (1.32) | 16 (0.68) | 2 (0.09) | 2,340 |
| 2017 | 40 (1.74) | 13 (0.56) | 0 | 2,303 |
| Total | 185 (1.19) | 120 (0.77) | 52 (0.33) | 15,547 |

**Figure C**


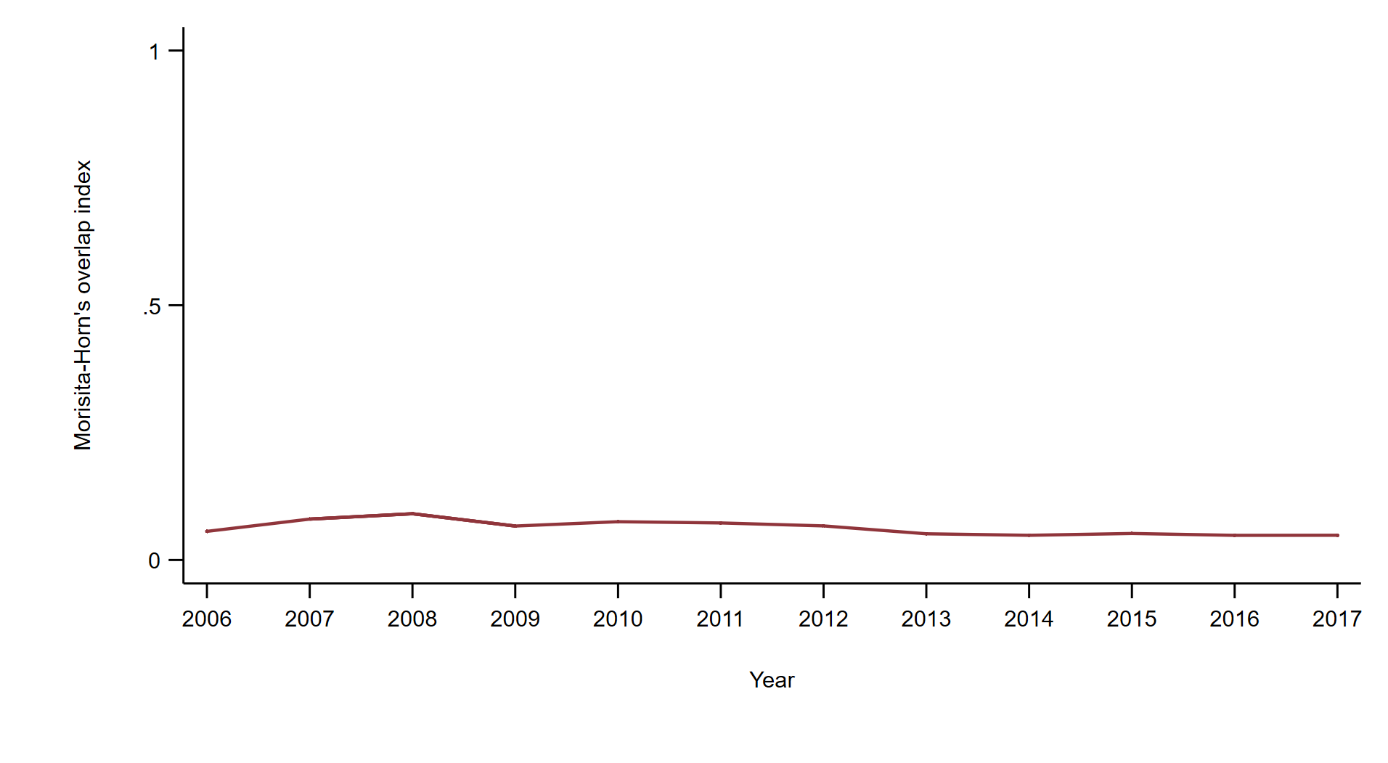


**Figure D**


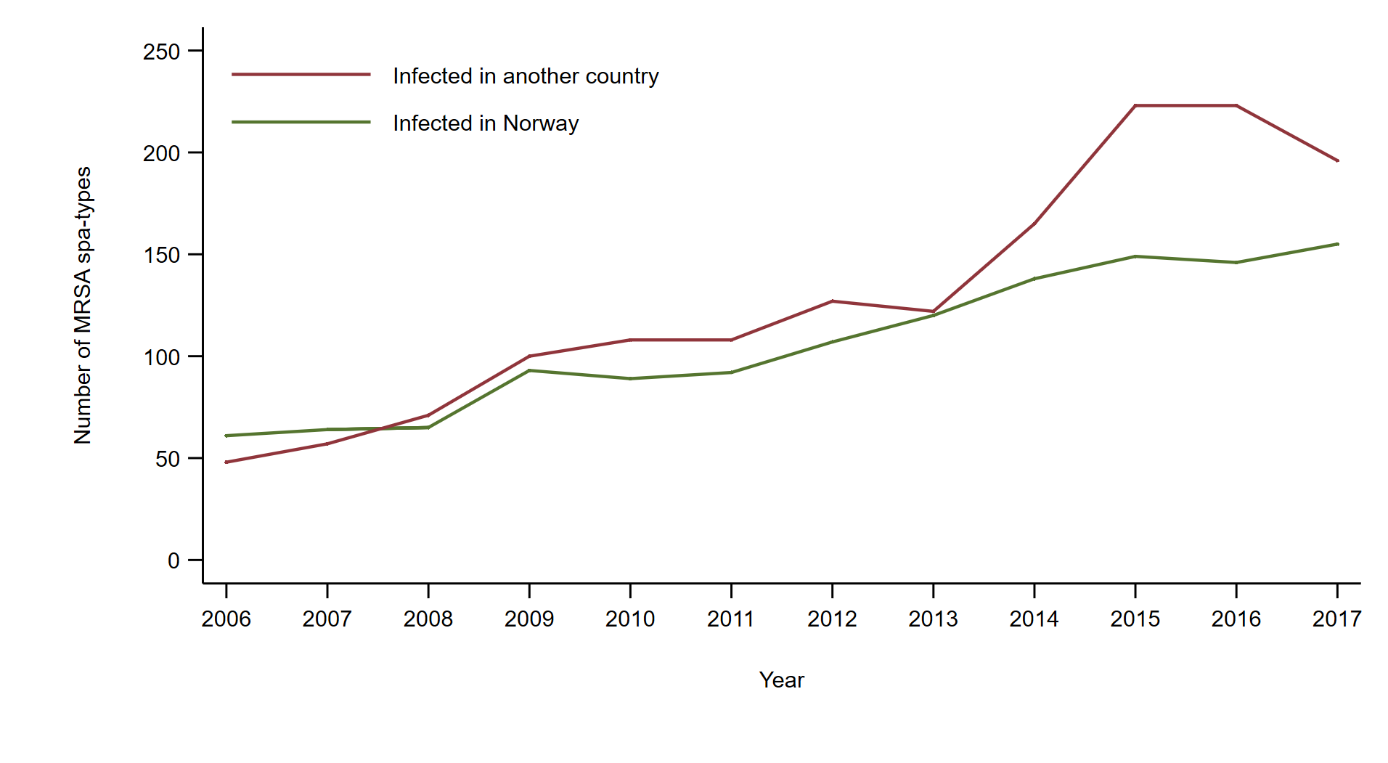


**Table C**

| **Codes** | **Explanations** |
| --- | --- |
| use "*dataset*.dta", clear | Opening the MSIS data file |
| keep if *diag*nose==1 & *persons*==1 & *variable*==1 | Selecting the subpopulation of interest:  *diagnose*: MRSA, VRE or CPO  *persons*==1: only unique individuals  *variable*: cases of interest e.g. infection, colonization, import, domestic, place of diagnosis |
| contract *year* *population* | Contracting the dataset to one record per year and three variables:  *year:* notification year (the year the sample was taken)  *population:* the population of Norway in each year  *_freq:* number of cases each year |
| sort *year* | Sorting the dataset by notification year |
| tsset *year,* yearly | Organizing the dataset to be time-series data, with year as the time unit |
| glm *_freq year*, exposure(*population*) family(nbinom ml) | Performing negative binomial regression in a generalized linear model using maximum likelihood optimization, and using number of cases per year as nominator and the annual population in Norway as denominator |
| predict resid, anscombe | Calculating the residuals after running the regression model |
| pac resid  ac resid | Checking to see if autocorrelation exists by producing correlograms with pointwise confidence intervals |
| glm *_freq year*, eform exposure(*population*) family(nbinom ml) | Performing the final regression model and reporting the coefficients as IRR |
